# Trazodone effects on developing brain

**DOI:** 10.1038/s41398-021-01217-w

**Published:** 2021-02-01

**Authors:** Zeljka Korade, Luke B. Allen, Allison Anderson, Keri A. Tallman, Thiago C. Genaro-Mattos, Ned A. Porter, Karoly Mirnics

**Affiliations:** 1 Department of Pediatrics, College of Medicine, University of Nebraska Medical Center, Omaha, 68198 NE USA; 2 Department of Biochemistry and Molecular Biology, College of Medicine, University of Nebraska Medical Center, Omaha, 68198 NE USA; 3 Munroe-Meyer Institute for Genetics and Rehabilitation, University of Nebraska Medical Center, Omaha, 68105 NE USA; 4 Department of Chemistry, Vanderbilt Institute of Chemical Biology and Vanderbilt Kennedy Center for Research on Human Development, Vanderbilt University, Nashville, 37235 TN USA

**Keywords:** Molecular neuroscience, Neuroscience

## Abstract

Trazodone (TRZ) is a commonly prescribed antidepressant with significant off-label use for insomnia. A recent drug screening revealed that TRZ interferes with sterol biosynthesis, causing elevated levels of sterol precursor 7-dehydrocholesterol (7-DHC). Recognizing the well-documented, disruptive effect of 7-DHC on brain development, we designed a study to analyze TRZ effects during pregnancy. Utilizing an in vivo model and human biomaterial, our studies were designed to also account for drug interactions with maternal or offspring *Dhcr7* genotype. In a maternal exposure model, we found that TRZ treatment increased 7-DHC and decreased desmosterol levels in brain tissue in newborn pups. We also observed interactions between *Dhcr7* mutations and maternal TRZ exposure, giving rise to the most elevated toxic oxysterols in brains of *Dhcr7*^*+/−*^ pups with maternal TRZ exposure, independently of the maternal *Dhcr7* genotype. Therefore, TRZ use during pregnancy might be a risk factor for in utero development of a neurodevelopmental disorder, especially when the unborn child is of *DHCR7*^*+/−*^ genotype. The effects of TRZ on 7-DHC was corroborated in human serum samples. We analyzed sterols and TRZ levels in individuals with TRZ prescriptions and found that circulating TRZ levels correlated highly with 7-DHC. The abundance of off-label use and high prescription rates of TRZ might represent a risk for the development of *DHCR7* heterozygous fetuses. Thus, TRZ use during pregnancy is potentially a serious public health concern.

## Introduction

The inhibition of 7-dehydrocholesterol reductase (DHCR7) enzyme, a key enzyme in the cholesterol biosynthesis pathway, leads to elevated levels of 7-dehydrocholesterol (7-DHC) and decreased levels of desmosterol and cholesterol^1^. Inhibition of DHCR7 is a side effect of multiple widely-used pharmaceuticals^2–4^. These compounds produce changes in sterol profiles that mimic biochemical changes arising from genetic mutations in humans (Supplemental Fig. 1)^5,6^. One of these compounds is trazodone (TRZ) hydrochloride (marketed under brand names Trazodone, Desyrel, Donaren, Molipaxin, Oleptro, Trazorel, and Trittico).

Approved by the FDA in 1981, TRZ is a potent 5-HT_2A_ and α_1_-adrenergic receptor antagonist and weak serotonin reuptake inhibitor^7^. TRZ and its major active metabolite *meta*-chlorophenylpiperazine (*m-*CPP) also bind to a variety of other receptors. The primary use of TRZ is the treatment of depression. However, TRZ has been extensively prescribed for off-label use as a treatment for insomnia^8,9^. In fact, the off-label use of TRZ for insomnia has surpassed its usage as an antidepressant^8^. Non-approved uses include treatment and/or self-treatment of opioid withdrawal symptom^10^, complex regional pain syndrome^11^, obsessive–compulsive disorder^12^, alcohol withdrawal^13–15^, schizophrenia^16^, dementia in Alzheimer’s disease, eating disorders, fibromyalgia, and erectile dysfunction^17^. Considering this extensive off-label use, it is not surprising that for the period from 2007 to 2017 there were a total of 226,057,791 TRZ prescriptions (Supplemental Fig. 2)^18^. The total number of TRZ prescriptions is almost twice as high as the number of oxycodone prescriptions.

Normal cholesterol homeostasis is essential for brain development, health, and life^19^. Genetic disruptions of the cholesterol biosynthesis pathway lead to various syndromes, including Smith–Lemli–Opitz syndrome, desmosterolosis, and chondrodysplasia punctata X-linked 2 (CDPX2)^5^. Our recent study examined levels of cholesterol and cholesterol precursors, desmosterol, and 7-DHC in blood samples of 123 psychiatric patients treated with various antipsychotic and antidepressant drugs and 85 healthy controls^20^. We found markedly increased circulating 7-DHC levels in patients treated with TRZ, suggesting that TRZ is a strong inhibitor of DHCR7^21^.

Knowing the well-documented, disruptive effect of 7-DHC on brain development^22–24^, and the widespread off-label use of TRZ in the human population, we designed a study to analyze TRZ effects during pregnancy using an in vivo model as well as a human biomaterial. Our studies were also designed to account for drug-genotype interactions, as we examined if the effects of TRZ were dependent on maternal or offspring *Dhcr7* genotype^25^. The study design is presented in Supplemental Fig. 3.

## Materials and methods

### Chemicals

Unless otherwise noted, all chemicals were purchased from Sigma-Aldrich Co (St. Louis, MO). High-performance liquid chromatography grade solvents were purchased from Thermo Fisher Scientific Inc. (Waltham, MA). TRZ was obtained from Sigma-Aldrich and dissolved in sterile DMSO solution for the experiments. All sterol standards, natural and isotopically labeled, used in this study are available from Kerafast, Inc. (Boston, MA).

### Trazodone injections in mice

Adult male and female B6.129P2(Cg)-*Dhcr7*^*tm1Gst*^/J stock # 007453 mice were purchased from Jackson Laboratories. Mice homozygous for the *Dhcr7*^*Ex8*^ allele lack the exon 8 coding sequence and flanking splice acceptor site of the targeted gene, resulting in the truncated DHCR7 mutation most frequently observed in SLOS patients (IVS8-1G > C). Homozygous mice die shortly after birth. Heterozygous *Dhcr7*^*+/*^^−^ mice are well, fertile, and indistinguishable from control, wild-type mice. Mice were maintained by breeding within the colony and refreshing twice a year with stock 000664 mice from Jackson Laboratories. Mice were housed under a 12 h light–dark cycle at constant temperature (25 °C) and humidity with ad libitum access to food (Teklad LM-485 Mouse/Rat Irradiated Diet 7912) and water in Comparative Medicine at the University of Nebraska Medical Center (UNMC), Omaha, NE. The time-pregnant female mice received i/p injections of vehicle (5% DMSO in saline) (VEH) or TRZ (20 mg/kg dissolved in VEH) from E12 to E19. In humans, TRZ (Desyrel) is given at a starting dose of 150 mg/day; and may be increased by 50 mg per day every 3–4 days to a maximum dose of 400 mg per day for outpatient use. If we take a typical dose of 150 mg/70 kg human body weight, this translates to 2.1 mg/kg/day. Animal Equivalent dose (AED in mg/kg) is calculated as AED (mg/kg) = human dose (mg/kg) (150 mg per day) × Km ratio (12.3) = 26.4 mg/kg^26^. As a result, we chose to use a low dose of 20 mg/kg in our mouse experiments, which translates back to 100–150 mg/day in humans, depending on the weight of the patient. Twelve WT and 12 *Dhcr7*^*+/*^^*−*^ female mice were used in our study. Five WT and seven HET mice were injected with VEH and four WT and eight HET mice were injected with TRZ. The mouse colony was monitored three times a day and all newborn pups (P0) were collected for dissection shortly after birth. The total number of pups analyzed for this study was 160 (see Table 1 for detailed description). Frozen brain tissue samples were sonicated in ice-cold PBS containing butylated hydroxytoluene (BHT) and triphenylphosphine (PPh_3_). The aliquots of homogenized tissue were used for sterol and oxysterol extractions and protein measurements. The protein was measured using a BCA assay (Pierce). Sterol levels were normalized to protein measurements and expressed as nmol/mg protein. All procedures were performed in accordance with the Guide for the Humane Use and Care of Laboratory Animals. The use of mice in this study was approved by the Institutional Animal Care and Use Committee of UNMC.

**Table 1:** Experimental groups.

**1**		**2**		**3**		**4**		**5**		**6**		**7**		**8**	
WT pup		HET pup		WT pup		HET pup		WT pup		HET pup		WT pup		HET pup	
14		23		15		14		25		18		27		24	
M	F	M	F	M	F	M	F	M	F	M	F	M	F	M	F
6	8	15	8	8	7	8	6	15	10	8	10	16	11	11	13
WT Mother								*Dhcr7*-HET Mother							
Vehicle				Trazodone				Vehicle				Trazodone			
*n* = 5				*n* = 4				*n* = 7				*n* = 8			

Groups 1–8 correspond to pups genotype. The numbers below WT and HET pups show how many pups were born and the next two rows show the distribution of male (M) and female (F) pups as determined by PCR. The pregnant mice were either WT or *Dhcr7*-HET. The numbers in the row below vehicle and TRZ show how many pregnant mice were used in the study. The total number of mice analyzed in this study were 160 pups from 12 vehicle-treated pregnant mice and 12 TRZ treated pregnant mice.

### Liquid chromatography–mass spectrometry (LC–MS)/MS (SRM) analyses

Sterols were extracted and derivatized with PTAD as described previously^27^ and placed in an Acquity UPLC system equipped with ANSI-compliant well plate holder coupled to a Thermo Scientific TSQ Quantis mass spectrometer equipped with an APCI source. Then 5 μL was injected onto the column (Phenomenex Luna Omega C18, 1.6 μm, 100 Å, 2.1 mm × 50 mm) with 100% MeOH (0.1% v/v acetic acid) mobile phase for 1.0 min runtime at a flow rate of 500 μL/min. Natural sterols were analyzed by selective reaction monitoring (SRM) using the following transitions: Chol 369 → 369, 7-DHC 560 → 365, desmosterol 592 → 560, lanosterol 634 → 602, with retention times of 0.7, 0.4, 0.3, and 0.3 min, respectively. SRMs for the internal standards were set to d_7_-Chol 376 → 376, d_7_-7-DHC 567 → 372, ^13^C_3_-desmosterol 595 → 563, ^13^C_3_-lanosterol 637 → 605. Final sterol numbers are reported as nmol/mg of protein.

### 7-DHC- and cholesterol-derived oxysterol analysis

7-DHC-derived oxysterols (DHCEO and 4α-OH-7-DHC) and cholesterol derived oxysterol 24-OH cholesterol were quantitated by LC-MS/MS using an APCI source in the positive ion mode. Lipid content from 200 μL of brain lysate was extracted and the neutral lipids fraction was purified by SPE chromatography as described previously^24^. Purified content was resuspended in methanol and 5 μL was injected onto the column (Phenomenex Luna Omega C18, 1.6 μm, 100 Å, 2.1 mm × 100 mm) using ACN (0.1% v/v acetic acid) (solvent A) and methanol (0.1% v/v acetic acid) (solvent B) as mobile phase. The gradient was: 5% **B** for 2 min; 5 to 95% **B** for 0.1 min; 95% **B** for 1.5 min; 95 to 5% **B** for 0.1 min; 5% **B** for 0.5 min. The oxysterols were analyzed by SRM using the following transitions: DHCEO 399 → 381, 4α-OH-7-DHC 383 → 365, and 24-OH cholesterol 367 → 367. The SRM for the internal standard (d_7_-chol) was set to 376 → 376 and response factors were calculated to accurately determine the oxysterol levels. Final oxysterol levels are reported as nmol/mg of protein.

### Human serum analysis

The office of Regulatory Affairs has determined that this study does not constitute human subject research as defined at 45CFR46.102(f). Serum samples were obtained from the Nebraska Biobank that is part of the Center for Clinical and Translational Research. The biobank contains de-identified residual serum samples from patients who consent to donate any left-over after the laboratory testing. Electronic Health Record personnel identified a group of de-identified 59 samples: from users of TRZ (25 samples) and age, sex, and race matched control (34 samples) group. LC–MS/MS analyses were performed to detect and quantify TRZ and its active metabolite *m*-CPP in samples with TRZ listed in the medical records. Serum TRZ and *m*-CPP extraction and serum drug measurements were performed as previously described^28^. Serum sterol measurements by LC–MS/MS was done as previously described^28^.

### Statistical analyses

Statistical analyses were performed using Graphpad Prism 8 for Windows. Data showed normal distribution, and the variance was comparable between the experimental and control groups. Unpaired two-tailed *t* tests were performed for individual comparisons between the two groups. Welch’s correction was employed when the variance between the two groups was significantly different. One-way ANOVA analyses were performed for comparisons among three or more groups. Two-way and three-way ANOVA analyses were performed to assess the interaction between maternal genotype, embryonic genotype, and drug treatment. The p values for statistically significant differences are highlighted in the figure legends.

## Results

### Maternal TRZ exposure alters cholesterol biosynthesis in the brain of offspring

WT and *Dhcr7*-HET pregnant mice received daily TRZ (20 mg/kg) or vehicle i/p injections from E12 to E19. This resulted in eight groups of newborn mice: (1) WT pup-WT mother + vehicle; (2) WT pup-WT mother + TRZ; (3) HET pup-WT mother + vehicle; (4) HET pup -WT mother + TRZ; (5) WT pup-HET mother + vehicle; (6) WT pup-HET mother + TRZ; (7) HET pup-HET mother + vehicle; and (8) HET pup-HET mother + TRZ (Table 1). We hypothesized that the most pronounced effect would be observed in the HET pups from HET mothers treated with TRZ (group 8).

**Fig. 1:**
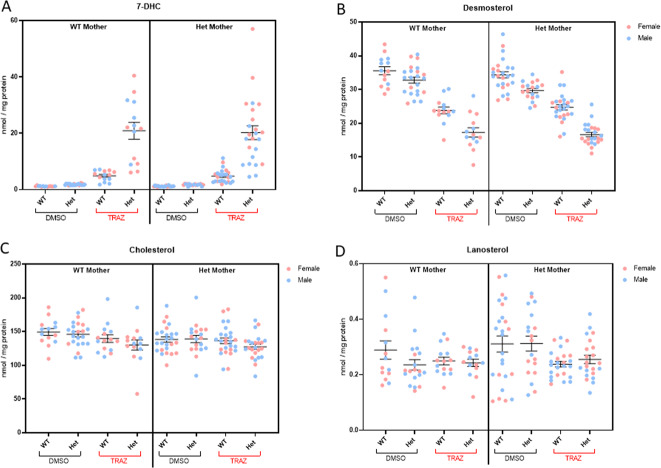
Maternal exposure to TRZ during pregnancy alters sterol profile in the brains of newborn pups. Pregnant WT and *Dhcr7*^*+/−*^ females were exposed to TRZ from E12 to E19 and pups’ brains were analyzed for sterols at P0. Each symbol corresponds to an individual pup brain; pink and blue symbols denote females and males, respectively. Bars correspond to the mean ± SEM. **A** 7-DHC; **B** Desmosterol; **C** Cholesterol; **D** Lanosterol. Statistical analysis: Table 2.

Brains were dissected from newborn pups and sterols were analyzed by LC–MS/MS. We report concentrations of cholesterol, desmosterol, 7-DHC, and lanosterol (Fig. 1) in WT and *Dhcr7*^*+/*^^−^ pups born to either WT or *Dhcr7*^*+/−*^ mothers. Maternal TRZ treatment, regardless of maternal or offspring *Dhcr7* genotype, led to significantly elevated 7-DHC in all groups (p < 0.001). In contrast, desmosterol was significantly decreased by TRZ treatment. The effects of TRZ on cholesterol levels were much less pronounced, though *Dhcr7*^*+/*^^−^ pups were more affected by TRZ than their WT littermates. Maternal genotype by itself did not have an effect on the TRZ-induced sterol changes. For most conditions, there was no statistically significant difference between females and males. The exception was 7-DHC where *Dhcr7*^*+/−*^ male pups from *Dhcr7*^*+/−*^ mothers were less affected than their female counterparts. Note that the greatest effects (and greatest variability) of TRZ on the sterol precursors were observed in the HET pups regardless of maternal genotype, suggesting a strong gene-treatment interaction in the newborn brain. This variability could not be explained by pup sex, or mouse genetic background. However, 7-DHC levels were significantly correlated with TRZ levels. Thus, we believe that the source of this variability is related to maternal TRZ metabolism, which might depend on food intake, activity, or other factors. Three-way ANOVA results examining the variables of treatment, maternal genotype, and pup genotype are presented in Table 2 corresponding to the data in Fig. 1.

**Table 2:** Three-way ANOVA of factors influencing brain sterol levels in newborn pups.

ANOVA table	7-DHC	Desmosterol	Cholesterol
Trazodone treatment^a^	***P*** < **0.0001**	***P*** < **0.0001**	***P*** = **0.005**
Maternal *Dhcr7* genotype^b^	*P* = 0.8237	*P* = 0.132	*P* = 0.0822
Pup *Dhcr7* genotype^b^	***P*** < **0.0001**	***P*** < **0.0001**	*P* = 0.1316
Trazodone * Maternal *Dhcr7* genotype	*P* = 0.8676	*P* = 0.09	*P* = 0.3774
Trazodone * Pup *Dhcr7* genotype	***P*** < **0.0001**	***P*** = **0.0093**	*P* = 0.2518
Maternal Dhcr7 genotype * Pup Dhcr7 genotype	*P* = 0.8658	*P* = 0.1991	*P* = 0.7382
Trazodone * Maternal *Dhcr7* genotype * Pup *Dhcr7* genotype	*P* = 0.8966	*P* = 0.9108	*P* = 0.7932

^a^TRZ versus DMSO alone.

^b^WT versus heterozygous for *DHCR7*.

Bold values indicates statistically significant.

### Maternal TRZ exposure greatly elevates 7-DHC derived oxysterols in the brain of offspring

**Fig. 2:**
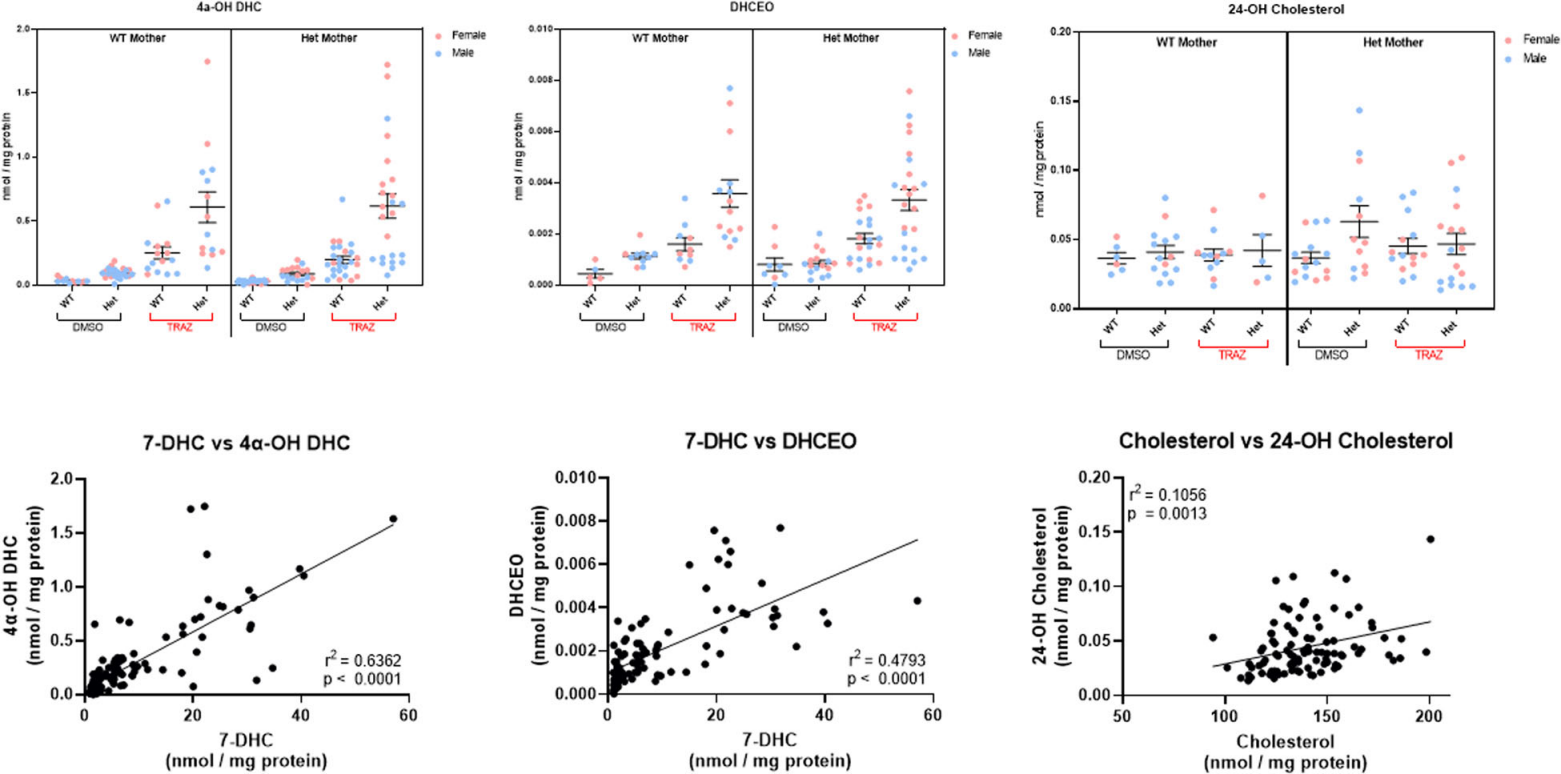
Maternal TRZ exposure greatly elevates 7-DHC derived oxysterols in the brains of offspring. P0 brain tissue of newborn pups was analyzed by LC–MS/MS to measure 7-DHC-derived oxysterols: **A** 4α-OH-DHC, **B** DHCEO, and cholesterol-derived oxysterol **C** 24-OH cholesterol. **D**, **E** Correlation of 7-DHC and 7-DHC derived oxysterols. **F** Correlation of cholesterol and cholesterol derived oxysterol. Each symbol corresponds to an individual pup brain; pink and blue symbols denote females and males, respectively. Statistical analysis: Table 3.

The experiment above revealed that *Dhcr7*^*+/−*^ pups from *Dhcr7*^*+/−*^ mothers have about 12 times more 7-DHC in the brain tissue than non-treated *Dhcr7*^*+/−*^ pups. 7-DHC is a highly unstable molecule, and spontaneously generates toxic oxysterols^22–24,29–31^. To determine if this pronounced elevation of highly oxidizable 7-DHC leads to the formation of 7-DHC derived oxysterols, we measured 4α-OH-DHC and DHCEO in brain tissue (Fig. 2). Maternal TRZ treatment, regardless of maternal or offspring *Dhcr7* genotype, led to significantly elevated 7-DHC-derived oxysterols in all groups receiving treatment. The largest increase in the level of oxysterols was observed in the brains of *Dhcr7*^*+/−*^ pups. Of the two oxysterols, 4α-OH-DHC was present at the highest levels. In addition, we found a strong correlation between the levels of 7-DHC and two 7-DHC-derived oxysterols (4α-OH-DHC: *r*^2^ = 0.6362; *P* < 0.0001; DHCEO: *r*^2^ = 0.4783; *P* < 0.0001). Three-way-ANOVA results examining the variables of treatment, maternal genotype, and pup genotype are presented in Table 3. In addition to 7-DHC-derived oxysterols, we assessed 24-OH-cholesterol levels derived from cholesterol. This oxysterol was not changed by TRZ treatment. The combined result show that the oxidative changes are primarily driven by 7-DHC levels (as opposed to cholesterol levels) and are most pronounced in the brains of maternally TRZ-treated HET pups regardless of maternal genotype.

**Table 3:** Three-way ANOVA of factors influencing brain oxysterol levels in newborn pups.

ANOVA table	4α-OH-DHC	DHCEO
Trazodone treatment^a^	***P*** < **0.0001**	***P*** < **0.0001**
Maternal *Dhcr7* genotype^b^	*P* = 0.7238	*P* = 0.9649
Pup *Dhcr7* genotype^b^	***P*** < **0.0001**	***P*** = **0.0004**
Trazodone * Maternal *Dhcr7* genotype	*P* = 0.8467	*P* = 0.9237
Trazodone * Pup *Dhcr7* genotype	***P*** < **0.0001**	***P*** = **0.019**
Maternal Dhcr7 genotype * Pup Dhcr7 genotype	*P* = 0.7059	*P* = 0.3433
Trazodone * Maternal *Dhcr7* genotype * Pup *Dhcr7* genotype	*P* = 0.7020	*P* = 0.8889

^a^TRZ versus DMSO alone.

^b^WT versus heterozygous for *DHCR7*.

### Circulating TRZ levels in human serum samples correlate with 7-DHC levels

**Fig. 3:**
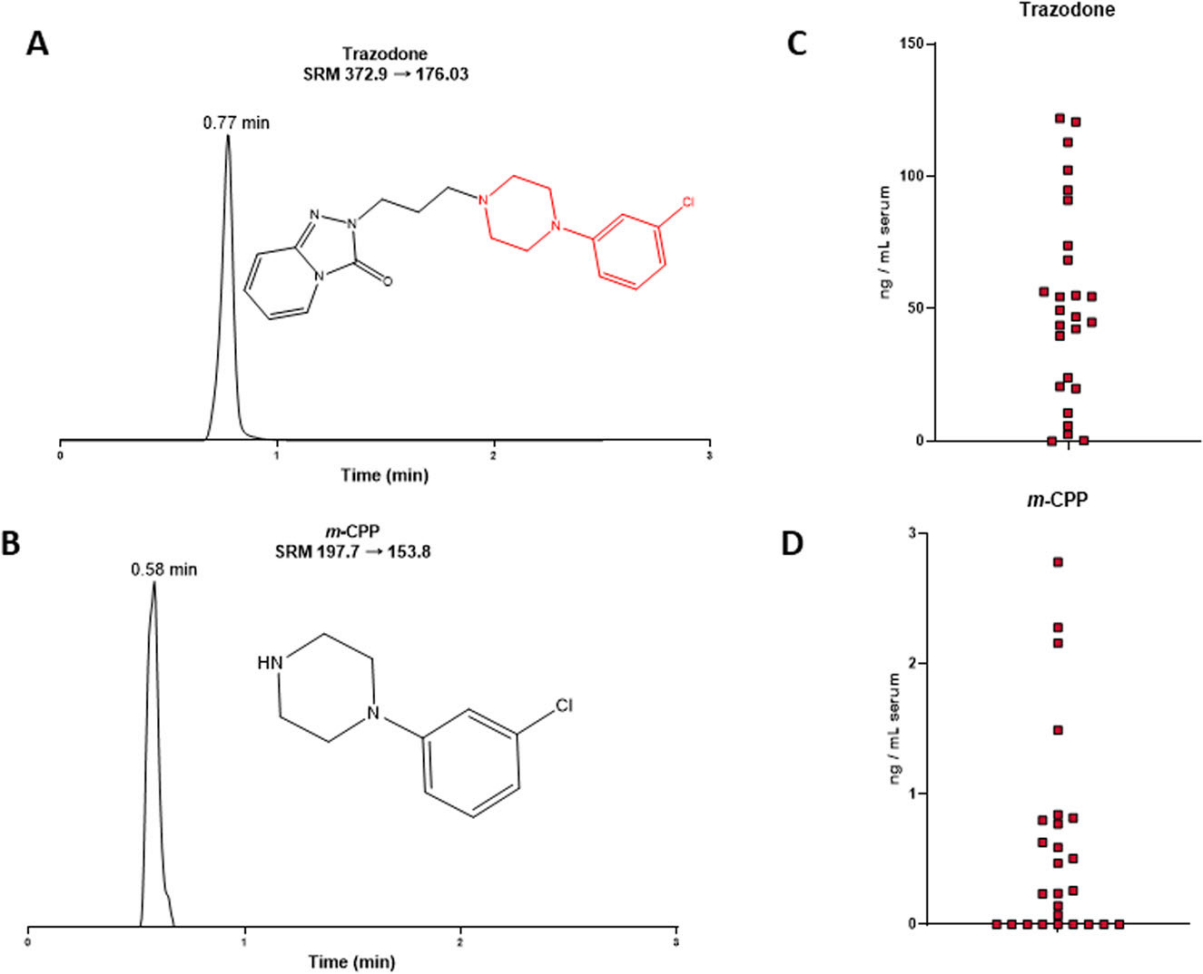
Analysis of human serum. A typical chromatogram and structure of **A** trazodone and **B** *m*CPP. **C** TRZ and **D** *m*CPP were present in measurable quantities in 25 samples that had TRZ listed in their medical records.

**Fig. 4:**
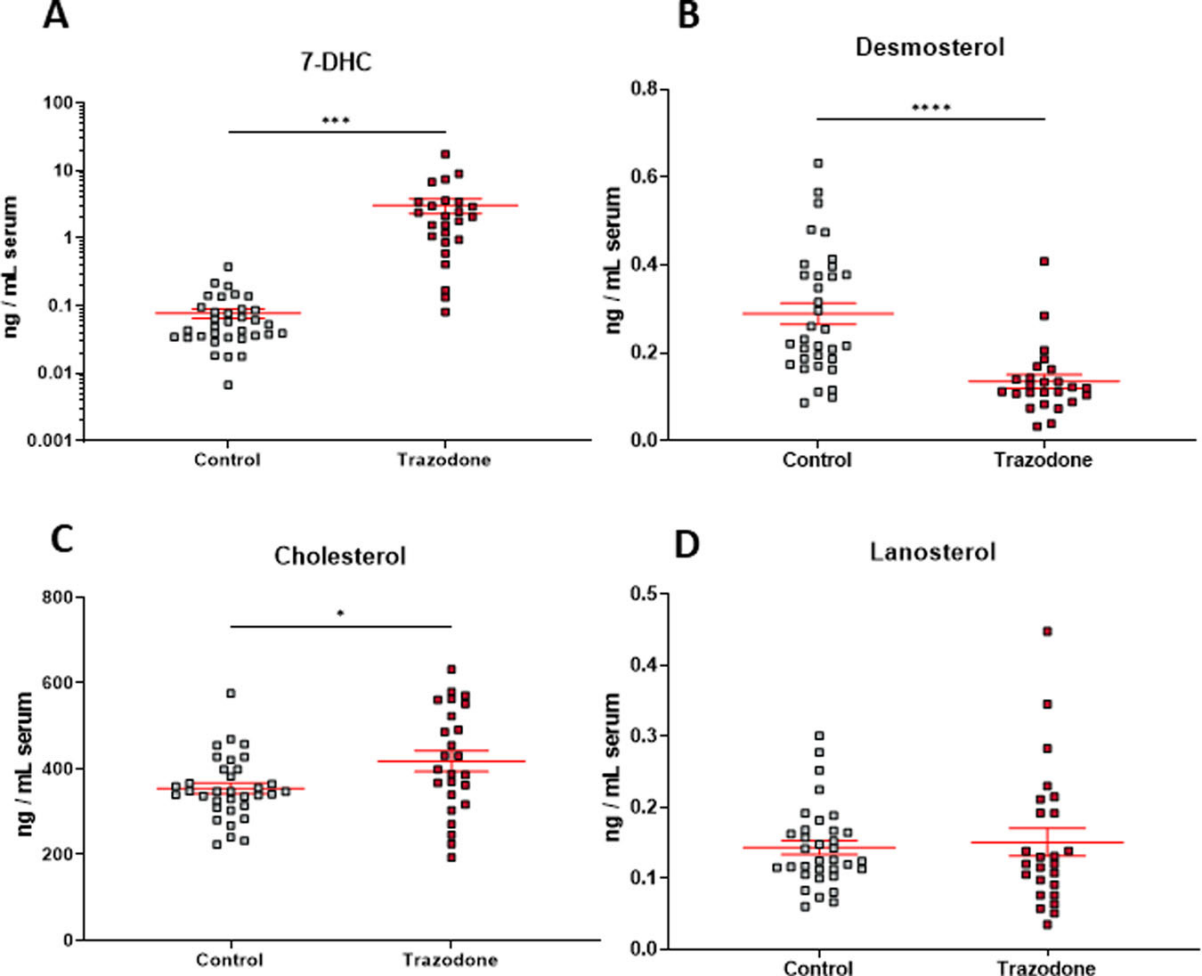
Sterol content in human serum. **A** 7-DHC, **B** desmosterol, **C** cholesterol, and **D** lanosterol were measured in serum samples from TRZ-treated individuals or controls. Note logarithmic *y*-axis to allow that all samples are visible as individual dots.

**Fig. 5:**
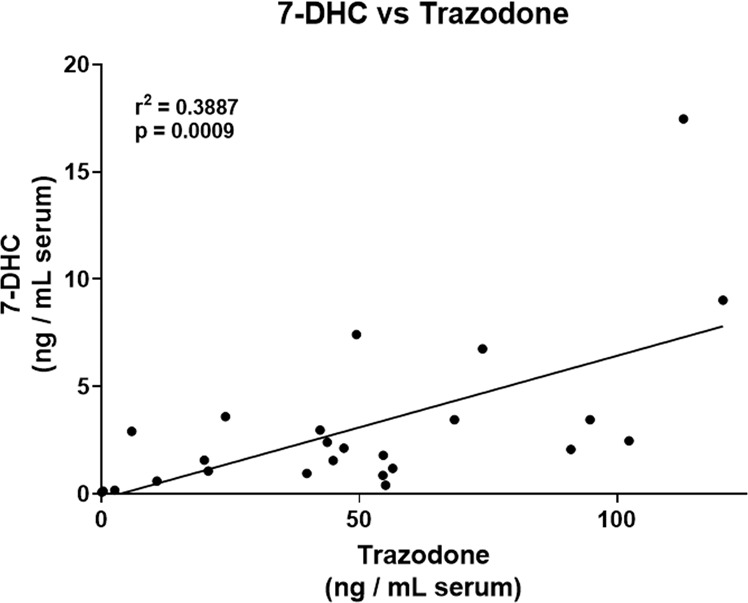
Correlation of sterol 7-DHC and TRZ in human serum. 7-DHC correlates with TRZ (*r*^2^ = 0.3887, *p* = 0.0009).

In our previous study, we showed that three drugs, aripiprazole, haloperidol, and TRZ, each increased circulating 7-DHC levels in blood samples of psychiatric patients^20^. To corroborate and expand on our previous observations related to TRZ, we analyzed sterols and TRZ levels in serum samples from individuals with TRZ prescriptions. TRZ and its active metabolite *m*-CPP were analyzed in 25 new serum samples (Fig. 3A–D) and compared to sterol levels in 34 age- and sex-matched control samples. Sterol measurements revealed that TRZ greatly elevated circulating levels of 7-DHC (*P* < 0.001) and decreased desmosterol (*P* < 0.001), with a small effect on cholesterol and no effect on lanosterol (Fig. 4A–D; Supplemental Fig. 4A–D male vs. females). The circulating TRZ levels correlated highly with 7-DHC (*r*^2^ = 0.3887; *P* = 0.0009) (Fig. 5). The outcome in this serum assessment suggests that TRZ-driven cholesterol biosynthesis changes in humans likely occur on a similar scale and by the same mechanisms as in our in vivo model and that it is likely occurring in multiple tissues outside the brain.

## Discussion

The key outcomes of our studies can be summarized as follows: (1) TRZ exposure increases 7-DHC, and decreases desmosterol levels in an in vivo rodent model. (2) *Dhcr7* mutations and maternal TRZ exposure interact, giving rise to toxic oxysterols that are disruptive to brain development. (3) The brains of *Dhcr7*^*+/−*^ pups show the greatest disruptions of sterol profile in response to maternal TRZ exposure, and this does not depend on maternal *Dhcr7* genotype. (4) TRZ exposure has a strong effect on circulating sterol levels in individuals taking TRZ. On a population scale, our findings point to a potentially serious public health impact of a medication as common as TRZ.

Elevation of 7-DHC concomitant with a decrease in desmosterol is characteristic for the inhibition of the DHCR7 enzyme (Supplemental Fig. 1). DHCR7 converts 7-DHC to cholesterol, and 7-dehydrodesmosterol into desmosterol. This accumulation of precursors has biological consequences, even when the difference is only a single double bond between the molecular structures^32^. 7-DHC is the most oxidizable (and unstable) sterol with a peroxidation rate constant of 2160, about 200-fold more than cholesterol and 10 times more than arachidonic acid, which is generally considered to be highly oxidizable^33^. As a result, 7-DHC readily gives rise to 7-DHC derived oxysterols^30^ that are well-described and their biological function has been extensively studied^22–24,30,31,34^. We have shown that 7-DHC derived oxysterols have potent and detrimental biological effects on cell proliferation, gene expression, and neuronal arborization^22–24,30,31,34^.

Over the last decade, many psychotropic drugs have been shown to interfere with sterol biosynthesis, increasing 7-DHC and resulting in oxysterol levels^2–4^. These properties were described initially in experiments with cell lines, followed by validation in animal models and human blood samples. Cariprazine^27,35^, haloperidol^20^, aripiprazole^36^, and TRZ are only a few examples of these DHCR7 inhibiting compounds. However, it should be noted that many other approved, non-CNS medications also show similar sterol-interfering effects, including amiodarone^28^. This raises an important question: which patient populations are most sensitive to the side effects of sterol-interfering drugs? Multiple animal studies to date suggest that two populations might be of risk—a developing embryo, and individuals with a single allele mutation in the DCHR7 gene. In addition, it appears that the risk is cumulative. In embryonic development, drug exposure and DHCR7 genotype interact, elevating 7-DHC to levels seen in genetic mouse models of SLOS. This brings us to the next question: the human relevance of our and others’ findings.

There is a legitimate concern that the inhibition of DHCR7 by TRZ in the developing human brain could have severe adverse effects. We base this view on the data from human genetic syndrome SLOS where mutations in DHCR7 affect CNS structure and function leading to developmental disabilities and autism^37,38^. Biochemical data from SLOS patient biomaterials suggests that TRZ could have teratogenic effects and should be avoided during pregnancy, especially if the fetus is heterozygous for *DCHR7*. Finally, a comprehensive literature review has identified that exposure to DHCR7 inhibitors during pregnancy leads to fetal loss in humans^39^, similar to those of known teratogens.

The public health relevance of our findings is also important to consider. Heterozygosity in *DHCR7* is quite common in the human population, with estimates ranging from 1 to 1.5%^40^, and it is not routinely tested for. Combined with the abundance of off-label use and high prescription rates, maternal TRZ exposure in *DHCR7* heterozygous fetuses might result in altered development, leading to developmental disability or other long-term consequences. It should be also considered that TRZ tends to be used in combination with other antipsychotics and pharmaceuticals that by themselves also inhibit sterol synthesis. The summative effect of this polypharmacy is unknown, but could be a significant concern: sterol homeostasis is undoubtedly essential for brain development and function, and interference with this finely tuned, intrinsically regulated system should be studied more extensively. Similarly, sterol biosynthesis disruption could have detrimental effects on other body systems, as sterols are essential precursors of many critical molecules—from steroid hormones to vitamins^1,5^.

## Supplementary information

Supplemental Figure 1

Supplemental Figure 2

Supplemental Figure 3

Supplemental Figure 4
