# Supplementary figures and images for: Trazodone effects on developing brain
Source: Transl Psychiatry. 2021 Feb 1;11:85. doi: 10.1038/s41398-021-01217-w (PMC7851398; doi:10.1038/s41398-021-01217-w)

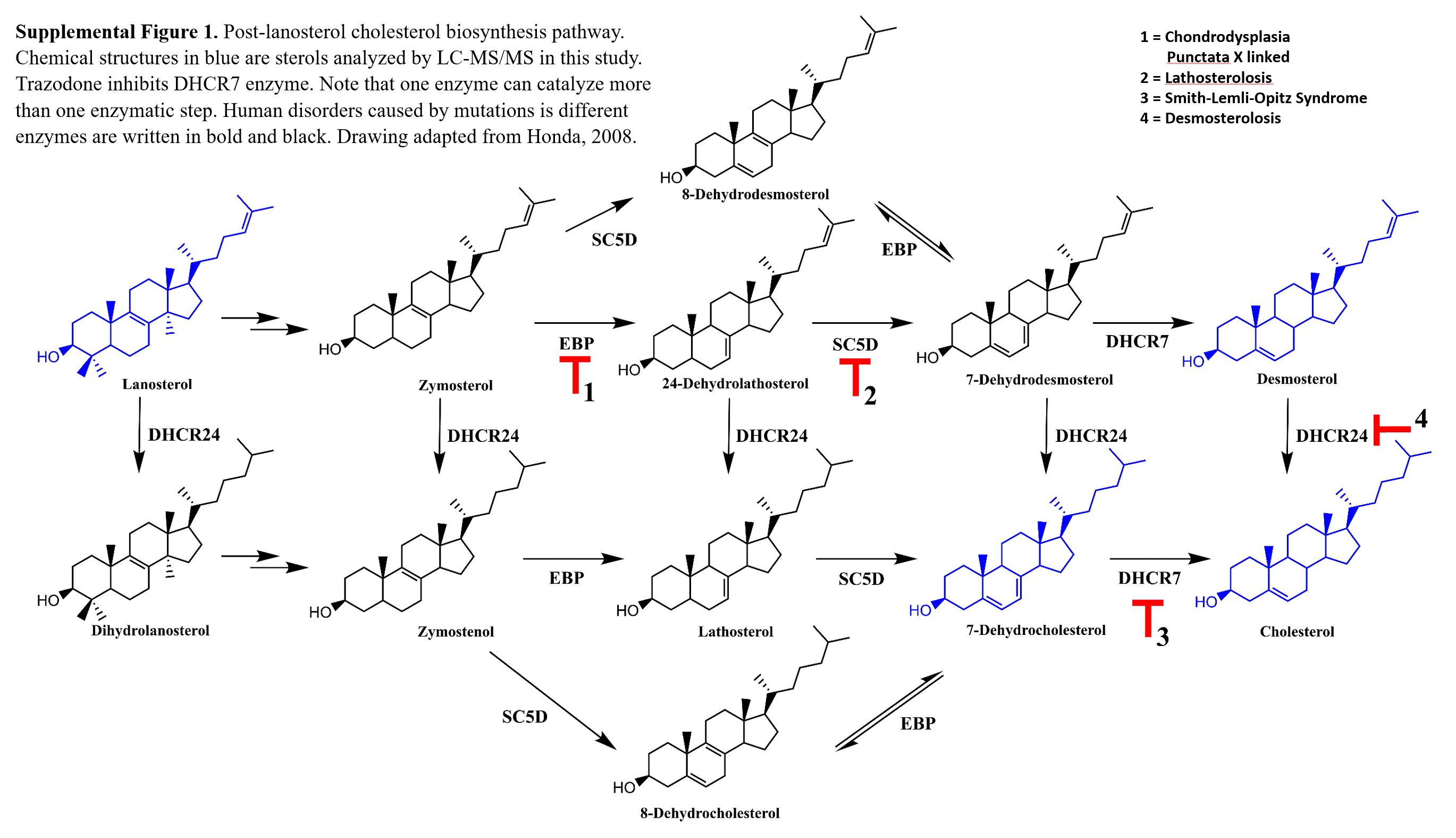

Supplement: Supplementary file 1 — Supplemental Figure 1 [file 41398_2021_1217_MOESM1_ESM.jpg]

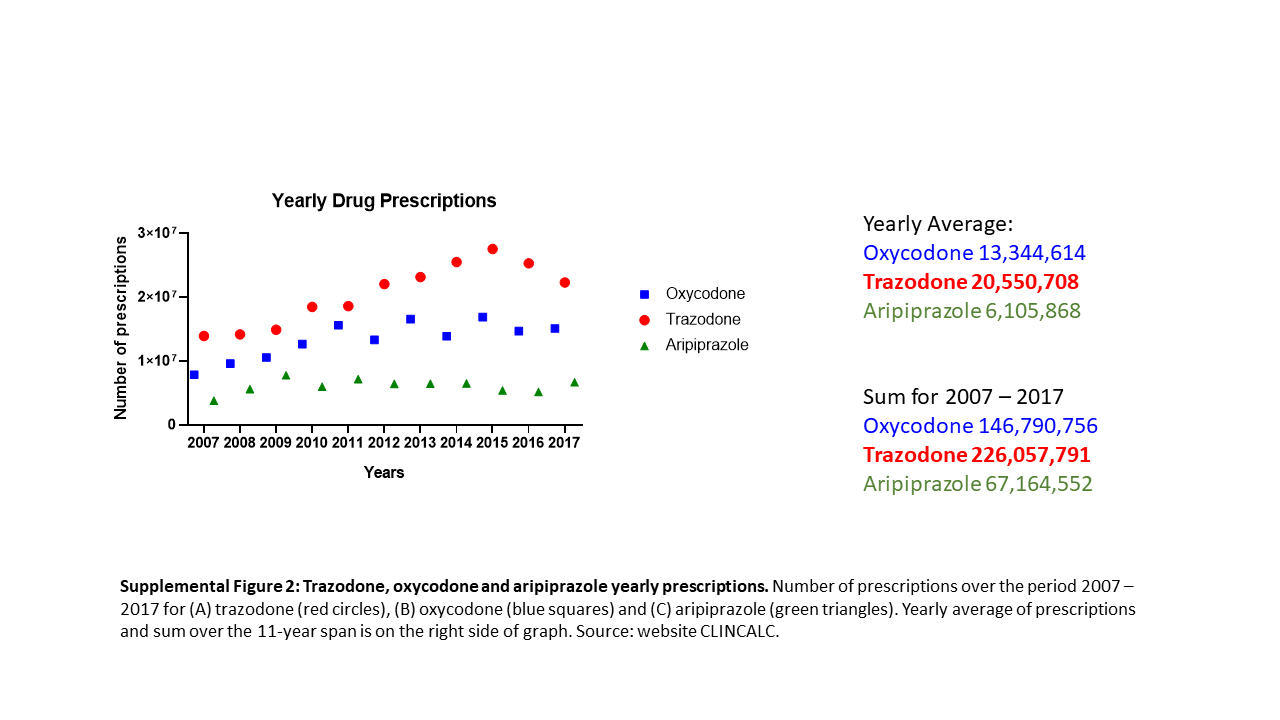

Supplement: Supplementary file 2 — Supplemental Figure 2 [file 41398_2021_1217_MOESM2_ESM.tif]

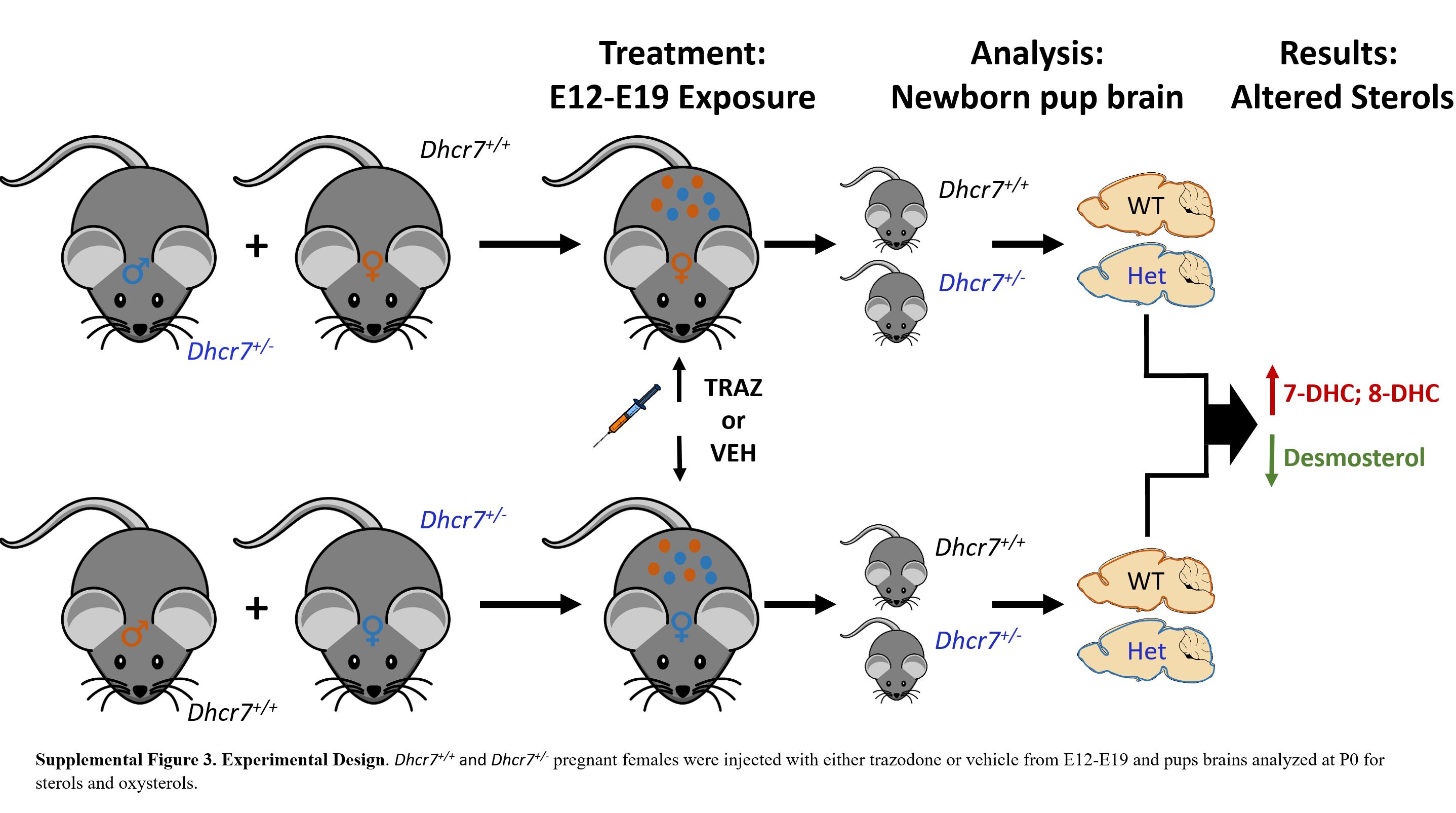

Supplement: Supplementary file 3 — Supplemental Figure 3 [file 41398_2021_1217_MOESM3_ESM.jpg]

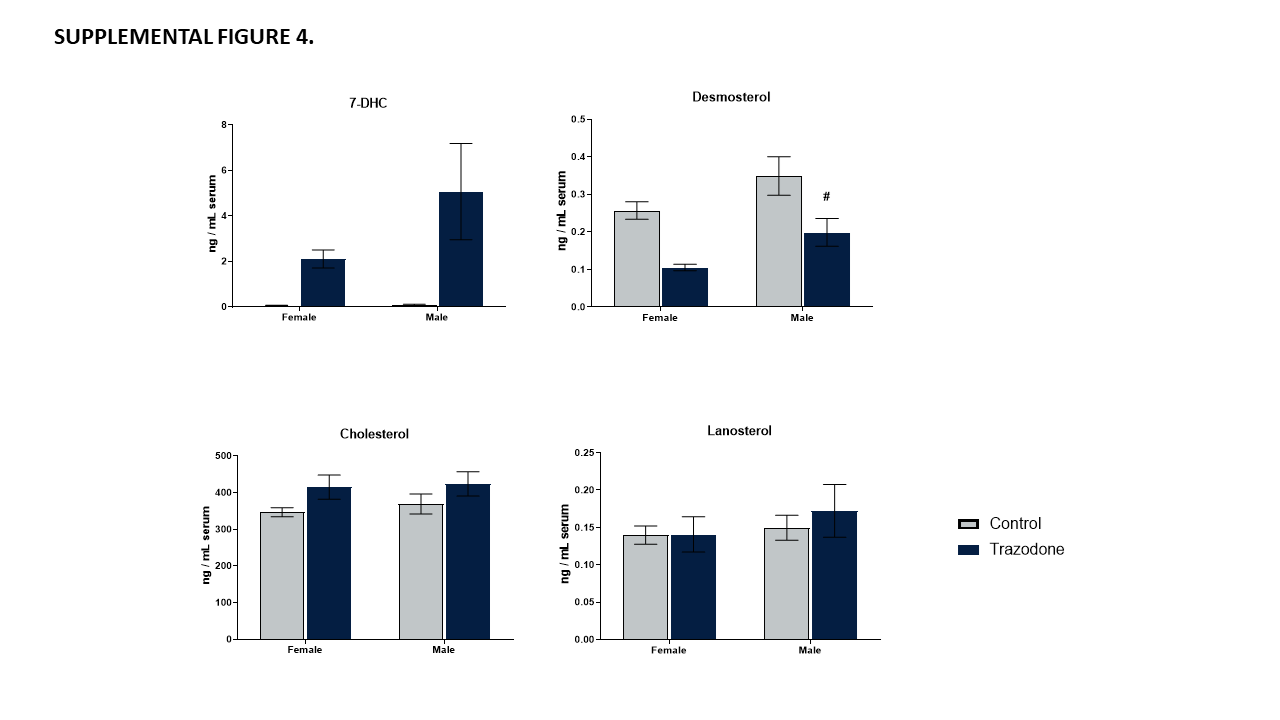

Supplement: Supplementary file 4 — Supplemental Figure 4 [file 41398_2021_1217_MOESM4_ESM.tif]
